# Path-dependency of energy decomposition analysis & the elusive nature of bonding†

**DOI:** 10.1039/d1cp04135e

**Published:** 2021-11-01

**Authors:** Jordi Poater, Diego M. Andrada, Miquel Solà, Cina Foroutan-Nejad

**Affiliations:** Departament de Química Inorgànica i Orgànica and IQTCUB, Universitat de Barcelona, Martí i Franquès 1-11 08028 Barcelona Catalonia Spain jordi.poater@ub.edu; ICREA, Pg. Lluís Companys 23 08010 Barcelona Spain; Faculty of Natural Sciences and Technology, Department of Chemistry, Saarland University 66123 Saarbrücken Germany diego.andrada@uni-saarland.de; Institut de Química Computacional i Catàlisi (IQCC) and Departament de Química, Universitat de Girona, C/Maria Aurèlia Capmany 69 17003 Girona Catalonia Spain miquel.sola@udg.edu; Institute of Organic Chemistry, Polish Academy of Sciences, Kasprzaka44/52, 01-224 Warsaw Poland cforoutan-nejad@icho.edu.pl

## Abstract

Here, we provide evidence of the path-dependency of the energy components of the energy decomposition analysis scheme, EDA, by studying a set of thirty-one closed-shell model systems with the *D*_2h_ symmetry point group. For each system, we computed EDA components from nine different pathways and numerically showed that the relative magnitudes of the components differ substantially from one path to the other. Not surprisingly, yet unfortunately, the most significant variations in the relative magnitudes of the EDA components appear in the case of species with bonds within the grey zone of covalency and ionicity. We further discussed that the role of anions and their effect on arbitrary Pauli repulsion energy components affects the nature of bonding defined by EDA. The outcome variation by the selected partitioning scheme of EDA might bring arbitrariness when a careful comparison is overlooked.

## Abstract 2

Here, we provide evidence of the path-dependency of the energy components of the energy decomposition analysis scheme, EDA, by studying a set of thirty-one closed-shell model systems with the *D*_2h_ symmetry point group.

## Graphical abstract

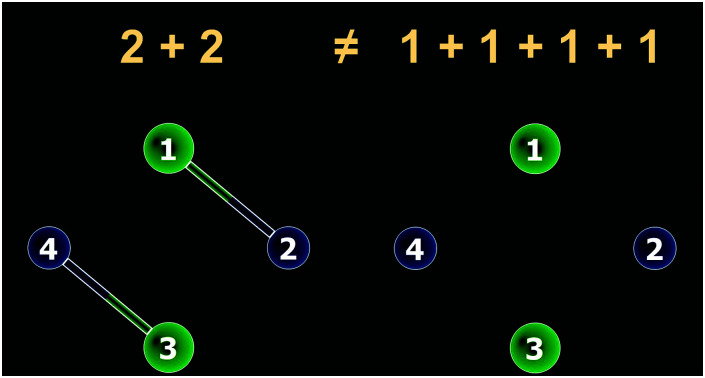

## Introduction

In a recent contribution, some of us discussed that the energy components of the energy decomposition analysis (EDA)^1–7^ method are – unlike the bond dissociation energy (*D*_e_) that is a state function – path (process) functions.^8^ The path-dependency of EDA is the result of the introduction of an arbitrary intermediate state in between the non-interacting fragments and the bonded molecule.^9^ Therefore, it is expected that the path-dependency of energy components to be a general problem among all EDA-based approaches and variants, irrespective of the number of additional energy components and presumptions implemented in the method.^10^ This may explain the discrepancies about the nature of chemical bonds assessed by EDA and other approaches.^11–15^ However, the gedankenexperiment that is suggested in the original paper to prove that the EDA components are path functions needs programming a new EDA code in which bond length can be controlled and the boundaries of the nearby atoms to be defined. However, the definition of the atoms in molecules when the distance between the interacting fragments is comparable to the equilibrium bond length has been a matter of debate in the chemical community.

A simpler alternative to test the path dependency of EDA energy components has been recently described by Solà *et al.* in the case of water tetramers.^16^ Briefly, they decomposed a water tetramer into four water molecules through seven different pathways. Summing up the contributions of the Pauli repulsion, electrostatic, and orbital interaction energies shows that each pathway leads to a slightly different contribution of the energy components while the sum over all components obtained from each pathway equals *D*_e_.^16^ Fortunately, the obtained data from different pathways showed no change in the general picture of bonding in water tetramer. Nonetheless, a raising question would be on the generalization of such observation: can different fragmentation schemes lead to a contradictory assessment of the nature of bonding in certain systems?

In this contribution we are not talking about the use of different fragments in EDA, but about different dissociation routes that lead to the same dissociated fragments. The fact that considering different fragments in EDA gives different results is well-known. For instance, if we analyze the chemical bond in LiF, considering Li^+^ and F^−^ as fragments, EDA indicates that covalency represents only 8% of stabilizing interactions. In contrast, if the fragments are chosen to be radicals, F˙ and Li˙, the covalency of LiF increases to 91%.^17^ One could wonder whether either the ionic or the radical fragmentation is the best option to discuss the bonding of LiF. Both fragmentations have arguments in favor. On the one hand, the radical fragments should be preferred because, for the gas-phase LiF molecule, the homolytic dissociation costs less energy than the heterolytic one, following the IUPAC recommendation of minimum-rupture energy.^18^ However, in the equilibrium geometry, the electronic distribution is closer to Li^+^ and F^−^ ions than to F˙ and Li˙ radicals. Using one or the other fragmentation scheme is a matter of choice and, in principle, both are acceptable, despite the results can differ enormously.

Herein, we show that even using the same fragments, either ionic or radical, if the order of fragmentation changes, the energy components of the EDA can also change, to prove the path (process) function of the EDA energy components. There is a reasonable consensus between different chemical bond theories on the nature of strong ionic or covalent bonds. However, the discrepancies often arise in two areas: (1) relative contributions of ionicity and covalency in bonds that are not pure covalent/ionic, and (2) the trends of the variation of ionicity/covalency in groups of closely related molecules. Here, we analyzed ionicity/covalency of bonds in 31 *D*_2h_ complexes with the general formula M_2_X_2_, where M represents a metal and X is a nonmetal. Our list includes species from both main group and transition metal elements as the following: Li_2_F_2_, Li_2_I_2_, Cs_2_I_2_, Be_2_X_2_ (X = O, S, Se, Te), Mg_2_X_2_ (X = O, S, Se), Ba_2_X_2_ (X = O, Se, Te), Ag_2_X_2_ (X = Cl, Br, I), B_2_X_2_ (X = N, P, As, Sb), Al_2_As_2_, Ga_2_X_2_ (X = N, P, As), In_2_X_2_ (X = N, P, As), Tl_2_N_2_, Tl_2_As_2_, Hg_2_O_2_, and Hg_2_S_2_.

## Results and discussion

**Fig. 1:**
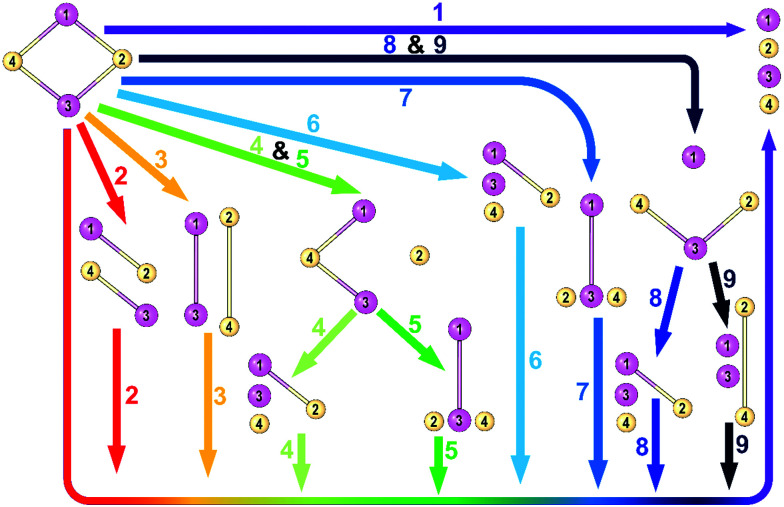
Schematic representation of nine pathways for breaking M_2_X_2_ into four isolated ions. Here, atoms 1 and 3 represent metal atoms, and atoms 2 and 4 denote nonmetal atoms.

Each complex is dissected into its closed-shell ionic components *via* nine different pathways as it is represented in Fig. 1. The energy components obtained from each pathway beside the equilibrium geometry of the studied species are provided in the ESI.† Pathway 1 is the direct decomposition of M_2_X_2_ compounds into four ions, two metal cations, and two nonmetal anions. Pathways 2 and 3 depict stepwise decomposition of the systems into two-atomic fragments and then decomposition of these fragments into isolated ions. Paths 4, 5, 8, and 9 represent 3-step decomposition into the ions and finally, paths 6 and 7 are two-step decomposition into 3 fragments and then four isolated ions. All fragments are considered in their lowest-lying singlet states.

**Fig. 2:**
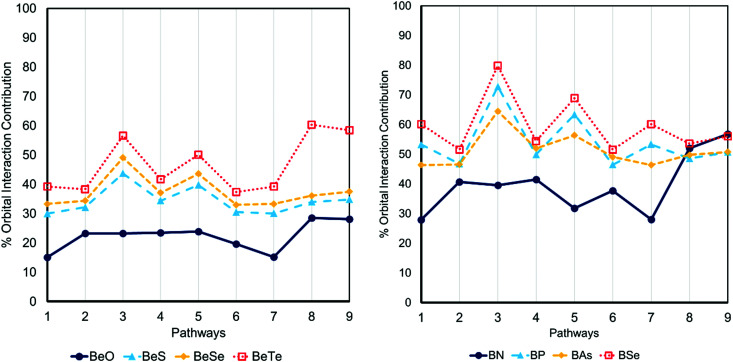
The percentage of the contribution of Δ*E*_oi_ in total interaction energy in dimers of beryllium chalcogenides and boron pnictogenides obtained from nine different pathways.

Orbital interaction energy, Δ*E*_oi_, is often associated with covalency within the framework of EDA. We start by examining the sensitivity of EDA in defining the nature of bonds to the selected pathway by measuring the contribution of Δ*E*_oi_ in beryllium chalcogenides. The interaction of the hard Be^2+^ ion with large and polarizable anions increases the relative contribution of the Δ*E*_oi_ in the total interaction energy as expected. However, as it is represented in Fig. 2, different pathways suggest various rates in the covalent character growth as the atomic numbers of the chalcogens increase. Interestingly, while in the case of Be_2_O_2_ pathways 2 to 5 predict nearly the same percentage of the Δ*E*_oi_ contribution in the interaction energy (23.3% to 23.9%), the same pathways predict significantly different covalent characters for heavier chalcogenides. Notably, the contribution of Δ*E*_oi_ in the Be–Te bond shows a significant oscillation varying between 43.4% to 62.6% of the total interaction energy.

The variation of the covalent character in boron pnictogenides is even more dramatic. In particular, while pathway 3 predicts that the covalency of B–Se is far more considerable than that of B–N bond, pathways 8 and 9 suggest that the covalency of B–N and B–Se are comparable and B–N is even more covalent than B–P and B–As bonds.

To further investigate the varying extent of the ionicity/covalency among M_2_X_2_ systems, the standard deviation of the percentage of electrostatic interaction energy, Δ*E*_elstat_, obtained from different pathways for each species is computed and listed in Table 1 along with the minimum, maximum, and the difference between the extremum values of the %Δ*E*_elstat_. The standard deviation and the difference between the minimum and maximum of the electrostatic contribution, Δ%Δ*E*_elstat_, obtained from different pathways are two key parameters that reflect the path-dependency of EDA energy components. The largest standard deviations are found for B_2_N_2_ (9.9), B_2_Sb_2_ (9.4), and B_2_P_2_ (8.7), respectively. The Δ%Δ*E*_elstat_ for 15 species are found to be more than 15% and those of 9 species are between 10–15%. The largest variations of the contribution of %Δ*E*_elstat_ and highest standard deviations belong to semimetals, systems with bonds that are indeed the borderline between ionic and covalent character.

**Table 1:** The standard deviation (SD), the minimum and maximum percentage of contribution of the Δ*E*_elstat_ to the interaction energy, and the difference between %Δ*E*_elstat_ max and %Δ*E*_elstat_ min for the studied systems

Molecules	SD	%Δ*E*_elstat_ min	%Δ*E*_elstat_ max	Δ%Δ*E*_elstat_	Molecules	SD	%Δ*E*_elstat_ min	%Δ*E*_elstat_ max	Δ%Δ*E*_elstat_
Li_2_F_2_	2.8	84.3	92.0	7.7	B_2_N_2_	9.9	43.2	72.1	28.8
Li_2_I_2_	3.4	70.2	80.3	10.0	B_2_P_2_	8.7	27.2	53.5	26.3
Cs_2_I_2_	2.3	84.0	90.2	6.2	B_2_As_2_	5.9	35.5	53.6	18.1
Be_2_O_2_	4.8	71.5	84.9	13.3	B_2_Sb_2_	9.4	20.1	48.4	28.3
Be_2_S_2_	4.7	56.3	70.0	13.7	Al_2_As_2_	5.5	58.7	73.2	14.5
Be_2_Se_2_	5.5	50.9	67.0	16.0	Ga_2_N_2_	7.9	63.5	84.9	21.4
Be_2_Te_2_	6.4	43.4	62.6	19.2	Ga_2_P_2_	5.6	58.5	72.7	14.2
Mg_2_O_2_	5.6	75.9	91.7	15.8	Ga_2_As_2_	5.7	57.5	71.9	14.4
Mg_2_S_2_	3.9	74.0	83.8	9.8	In_2_N_2_	8.3	36.6	64.2	27.6
Mg_2_Se_2_	4.1	69.7	81.0	11.3	In_2_P_2_	5.9	61.5	76.8	15.4
Ba_2_O_2_	7.5	64.2	85.8	21.6	In_2_As_2_	6.1	59.5	75.9	16.4
Ba_2_Se_2_	4.1	74.8	86.7	11.9	Tl_2_N_2_	8.0	32.8	60.2	27.4
Ba_2_Te_2_	3.9	74.7	85.6	10.9	Tl_2_As_2_	6.6	58.2	76.2	18.0
Ag_2_Cl_2_	2.8	71.7	80.9	9.2	Hg_2_O_2_	5.4	65.3	81.7	16.5
Ag_2_Br_2_	2.7	70.4	79.3	8.9	Hg_2_S_2_	4.3	66.4	78.2	11.8
Ag_2_I_2_	2.8	67.8	76.4	8.6					

We may set an arbitrary boundary between the ionic and covalent bonds, for instance, we can agree that an electrostatic contribution of less than 50% characterizes a covalent (polar covalent) bond while a contribution of more than 50% denotes an ionic bond (with polarized ions). The nature of the bonding in six species (Be_2_Te_2_, B_2_N_2_, B_2_P_2_, B_2_As_2_, In_2_N_2_, and Tl_2_N_2_) changes by changing the pathways to assess the energy components of EDA because the %Δ*E*_elstat_ in these species changes from below 50% to more than 50%. The largest variation of %Δ*E*_elstat_ is found for B_2_N_2_ (Δ%Δ*E*_elstat_ = 28.8) that also shows the largest standard deviation (9.9) upon changing the pathways for EDA analysis. B_2_Sb_2_ has the second-largest variations in the standard deviation and electrostatic contribution in the interaction energy. However, this molecule remains within the arbitrary realm of covalent bonds as %Δ*E*_elstat_ of the system changes from 20.1% to 48.4% that is within the limit of covalency.

The most significant variations in the Δ%Δ*E*_elstat_ values are observed in the case of species containing triply charged anions, in particular nitride. The same species have the largest standard deviation in the values of their Pauli repulsion values, Table S2 (ESI†). In fact, a balance between these two energy components, which are defined at the first steps of the EDA approach, defines the magnitude of the orbital interaction energy and therefore the covalent character of the molecule. The fragmentation paths involving dissociation of highly charged anions in their early stages, *e.g.* paths 4 and 5, result in significantly larger Pauli repulsion components than the paths in which cations are dissociated first, *e.g.* paths 8 and 9, Table S2 (ESI†). This suggests that the magnitude of the arbitrary Pauli repulsion can be controlled at will. During a hypothetical stepwise EDA, partial relaxation of the wave function as discussed before,^8^ has the same effect on the magnitude of the Pauli repulsion and consequently on the rest of the EDA energy components. This conclusion is further supported by the fact that two seemingly different paths, 1 and 7, in which anions are dissociated at the same time in the first step, provide nearly identical energy components.

## Conclusions

In summary, our analysis reveals that the classification of the chemical bond into ionic or covalent in the studied systems based on EDA results can change depending on the choice of the pathways. This issue is, in particular, more pronounced in the case of systems that have mixed ionic-covalent character. Taking the arbitrary border between the ionic and covalent bonds as 50%, there is a transition from the realm of ionicity to covalency with the change of the selected pathways to perform EDA in the case of six systems (Be_2_Te_2_, B_2_P_2_, B_2_P_2_, B_2_As_2_, In_2_N_2_, and Tl_2_N_2_) among the thirty-one studied. Besides, the nature of bonds in a number of systems varies significantly from ionic to strongly polar covalent like B–Sb bonds.

## Methods

All DFT calculations were carried out using the Amsterdam Density Functional (ADF) software. Geometries were optimized without any constraints using ZORA-BLYP/TZ2P level of theory. Vibrational frequency analysis was performed for all optimized species to confirm that were local minima.^19–21^

In the Energy Decomposition Analysis method, the dissociation energy in molecule AB is decomposed into:

1 *−D*_e_ = Δ*E*_prep_ + Δ*E*_int_

In this formula, the preparation energy Δ*E*_prep_ (also referred to as deformation or strain energy) is the amount of energy required to deform two individual (isolated) fragments A and B from their equilibrium structure to the geometry that they acquire in the overall AB molecule and to bring them to their reference electronic states. The interaction energy, Δ*E*_int_, corresponds to the actual energy change when these geometrically deformed and electronically prepared fragments are combined to form molecule AB. It is analyzed in the framework of the Kohn–Sham Molecular Orbital (MO) model using a quantitative decomposition of the interaction into electrostatic, Pauli repulsion (or exchange repulsion), and orbital interactions:

2 Δ*E*_int_ = Δ*E*_elstat_ + Δ*E*_Pauli_ + Δ*E*_oi_ (+Δ*E*_disp_)

The instantaneous interaction energy Δ*E*_int_ between two fragments A and B in a molecule AB is partitioned into three terms, namely, (1) the quasiclassical electrostatic interaction Δ*E*_elstat_ between the fragments; (2) the repulsive exchange (Pauli) interaction Δ*E*_Pauli_ between electrons of the two fragments having the same spin, and (3) the orbital (covalent) interaction Δ*E*_oi_ which comes from polarization and orbital mixing between the fragments. The latter term can be decomposed into contributions of orbitals with different symmetry Γ, which makes it possible to distinguish between *σ*, *π*, and *δ* contributions to bonding (Δ*E*_oi_ = ΣΔ*E*_*Γ*_). Δ*V*_elstat_ and Δ*E*_oi_ are associated with covalent and ionic contributions to the bonding, respectively.^5–7,22,23^ Finally, if the density functional used in the calculations contains dispersion corrections (not in our case), then in eqn (2) there is another term, Δ*E*_disp_, that takes into account the interactions due to dispersion forces.

## Conflicts of interest

The authors declare no competing financial interest.

## Supplementary Material

CP-024-D1CP04135E-s001
