# Supplementary material for: Path-dependency of energy decomposition analysis & the elusive nature of bonding
Source: Phys Chem Chem Phys. 2021 Nov 1;24(4):2344–8. doi: 10.1039/d1cp04135e (PMC8790740; doi:10.1039/d1cp04135e)
Supplement: CP-024-D1CP04135E-s001 [file CP-024-D1CP04135E-s001.pdf]

Electronic Supplementary Material (ESI) for Physical Chemistry Chemical Physics.
This journal is © the Owner Societies 2021

Electronic Supplementary Information

Path-Dependency of Energy Decomposition Analysis & the Elusive Nature of Bonding

Jordi Poater[1,2*], Diego M. Andrada[3*], Miquel Solà[4*], Cina Foroutan-Nejad[5*]

[1] *Departament de Química Inorgànica i Orgànica and IQTCUB, Universitat de Barcelona, Martí i Franquès 1-11, 08028 Barcelona, Catalonia, Spain*

[2] *ICREA, Pg. Lluís Companys 23, 08010 Barcelona, Spain*[3] *Faculty of Natural Sciences and Technology, Department of Chemistry, Saarland University, 66123 Saarbrücken, Federal Republic of Germany.*

[4] *Institut de Química Computacional i Catàlisi (IQCC) and Departament de Química, Universitat de Girona, C/ Maria Aurèlia Capmany 69, 17003 Girona, Catalonia, Spain*

[5] *Institute of Organic Chemistry, Polish Academy of Sciences, Kasprzaka44/52, 01-224, Warsaw, Poland*

Corresponding Author's Emails:

Jordi Poater: jordi.poater@ub.edu

Diego M. Andrada: *diego.andrada@uni-saarland.de*

Miquel Solà: miquel.sola@udg.edu

Cina Foroutan-Nejad: cforoutan-nejad@icho.edu.pl

Table S1. Cartesian coordinates (in Å) and electronic energy (in kcal mol^{-1}) of all M_2X_2 systems under analysis at the ZORA-BLYP/TZ2P level of theory.

Li_2F_2 (-378.10)

1.Li	0.000000	1.112316	0.000000
2.F	0.000000	0.000000	-1.337827
3.F	0.000000	0.000000	1.337827
4.Li	0.000000	-1.112316	0.000000

Li_2I_2 (-224.03)

1.Li	0.000000	1.393888	0.000000
2.I	0.000000	0.000000	-2.197345
3.Li	0.000000	-1.393888	0.000000
4.I	0.000000	0.000000	2.197345

Cs_2I_2 (-195.58)

1.Cs	0.000000	2.476692	0.000000
2.I	0.000000	0.000000	-2.649959
3.Cs	0.000000	-2.476692	0.000000
4.I	0.000000	0.000000	2.649959

Be_2O_2 (-473.18)

1.Be	0.000000	0.864583	0.000000
2.O	0.000000	0.000000	-1.211816
3.Be	0.000000	-0.864583	0.000000
4.O	0.000000	0.000000	1.211816

Be_2S_2 (-327.76)

1.Be	0.000000	0.966901	0.000000
2.S	0.000000	0.000000	-1.667858
3.Be	0.000000	-0.966901	0.000000
4.S	0.000000	0.000000	1.667858

Be_2Se_2 (-289.51)

1.Be	0.000000	0.996091	0.000000
2.Se	0.000000	0.000000	-1.807054
3.Be	0.000000	-0.996091	0.000000
4.Se	0.000000	0.000000	1.807054

Be_2Te_2 (-239.03)

1.Be	0.000000	1.034713	0.000000
2.Te	0.000000	0.000000	-2.033021
3.Be	0.000000	-1.034713	0.000000
4.Te	0.000000	0.000000	2.033021

Mg_2O_2 (-334.76)

1.Mg	0.000000	1.208250	0.000000
2.O	0.000000	0.000000	-1.448050
3.Mg	0.000000	-1.208250	0.000000
4.O	0.000000	0.000000	1.448050

Mg_2S_2 (-237.42)

1.Mg	0.000000	1.325569	0.000000
2.S	0.000000	0.000000	-1.923848
3.Mg	0.000000	-1.325569	0.000000
4.S	0.000000	0.000000	1.923848

Mg_2Se_2 (-212.19)

1.Mg	0.000000	1.356781	0.000000
2.Se	0.000000	0.000000	-2.057708
3.Mg	0.000000	-1.356781	0.000000
4.Se	0.000000	0.000000	2.057708

Ba_2O_2 (-431.80)

1.Ba	0.000000	1.758860	0.000000
2.O	0.000000	0.000000	-1.457328
3.Ba	0.000000	-1.758860	0.000000
4.O	0.000000	0.000000	1.457328

Ba_2Se_2 (-291.63)

1.Ba	0.000000	2.060208	0.000000
2.Se	0.000000	0.000000	-2.113937
3.Ba	0.000000	-2.060208	0.000000
4.Se	0.000000	0.000000	2.113937

Ba_2Te_2 (-251.89)

1.Ba	0.000000	2.140377	0.000000
2.Te	0.000000	0.000000	-2.372857
3.Ba	0.000000	-2.140377	0.000000
4.Te	0.000000	0.000000	2.372857

B_2N_2 (-450.31)

1.B	0.000000	1.506885	0.000000
2.N	0.000000	0.000000	-0.648131
3.B	0.000000	-1.506885	0.000000
4.N	0.000000	0.000000	0.648131

B_2P_2 (-375.65)

1.B	-0.795110	0.006147	0.007309
2.P	0.004919	-1.674785	0.022881
3.P	0.007423	1.687342	-0.007255
4.B	0.807768	0.006296	0.008319

B_2As_2 (-310.58)

1.B	0.000000	1.005312	0.000000
2.As	0.000000	0.000000	-1.688464
3.B	0.000000	-1.005312	0.000000
4.As	0.000000	0.000000	1.688464

B_2Sb_2 (-284.14)

1.B	0.000000	0.883559	0.000000
2.Sb	0.000000	0.000000	-1.964706
3.B	0.000000	-0.883559	0.000000
4.Sb	0.000000	0.000000	1.964706

Al_2As_2 (-253.93)

1.Al	0.207997	0.432852	1.640815
2.As	-1.253733	-0.568107	0.000000
3.As	1.852247	-0.226870	0.000000
4.Al	0.207997	0.432852	-1.640815

Ga_2N_2 (-329.27)

1.Ga	-1.509689	0.000000	0.020413
2.N	0.000000	-1.155901	-0.000158
3.Ga	1.509689	0.000000	0.020413
4.N	0.000000	1.155901	-0.000158

Ga_2P_2 (-206.19)

1.Ga	0.000000	1.505328	0.000000
2.P	0.000000	0.000000	-1.838686
3.Ga	0.000000	-1.505328	0.000000
4.P	0.000000	0.000000	1.838686

Ga_2As_2 (-192.07)

1.Ga	0.000000	1.548680	0.000000
2.As	0.000000	0.000000	-1.934219
3.Ga	0.000000	-1.548680	0.000000
4.As	0.000000	0.000000	1.934219

In_2N_2 (-396.03)

1.In	-2.395882	0.000000	0.010127
2.N	0.000000	-0.607592	0.010127
3.In	2.395882	0.000000	0.010127
4.N	0.000000	0.607592	0.010127

In_2P_2 (-214.13)

1.In	0.000000	1.677216	0.000000
2.P	0.000000	0.000000	-1.987447
3.In	0.000000	-1.677216	0.000000
4.P	0.000000	0.000000	1.987447

In_2As_2 (-204.02)

1.In	0.000000	1.710666	0.000000
2.As	0.000000	0.000000	-2.079756
3.In	0.000000	-1.710666	0.000000
4.As	0.000000	0.000000	2.079756

Tl_2N_2 (-390.83)

1.Tl	-2.575736	0.000000	0.010127
2.N	0.000000	-0.593661	0.010127
3.Tl	2.575736	0.000000	0.010127
4.N	0.000000	0.593661	0.010127

Tl_2As_2 (-184.61)

1.Tl	0.000000	2.106571	0.000000
2.As	0.000000	0.000000	-1.702548
3.Tl	0.000000	-2.106571	0.000000
4.As	0.000000	0.000000	1.702548

Ag_2Cl_2 (-185.88)

1.Ag	0.000000	1.426227	0.000000
2.Cl	0.000000	0.000000	-2.105713
3.Cl	0.000000	0.000000	2.105713
4.Ag	0.000000	-1.426227	0.000000

Ag_2Br_2 (-172.40)

1.Ag	0.000000	1.417299	0.000000
2.Br	0.000000	0.000000	-2.246899
3.Br	0.000000	0.000000	2.246899
4.Ag	0.000000	-1.417299	0.000000

Ag_2I_2 (-156.08)

1.Ag	0.000000	1.408121	0.000000
2.I	0.000000	0.000000	-2.432020
3.I	0.000000	0.000000	2.432020
4.Ag	0.000000	-1.408121	0.000000

Hg_2O_2 (-137.68)

1.Hg	0.000000	1.449251	0.000000
2.O	0.000000	0.000000	-1.676602
3.Hg	0.000000	-1.449251	0.000000
4.O	0.000000	0.000000	1.676602

Hg_2S_2 (-101.59)

1.Hg	0.000000	1.457218	0.000000
2.S	0.000000	0.000000	-2.101081
3.Hg	0.000000	-1.457218	0.000000
4.S	0.000000	0.000000	2.101081

Table S2. Energy decomposition analysis data (in kcal mol^{-1}) for the 9 different paths (Figure 1) of all M_2X_2 systems under analysis at the ZORA-BLYP/TZ2P level of theory. Standard deviation is also enclosed (in italics).

Li_2F_2

paths	ΔE_{int}	ΔE_{Pauli}	ΔE_{elstat}	ΔE_{oi}	*%elstat*	*%oi*
**Path 1**	-445.5	85.6	-488.7	-42.4	*92.0*	*8.0*
**Path 2**	-445.5	93.8	-470.3	-69.0	*87.2*	*12.8*
**Path 3**	-445.5	72.1	-439.1	-78.5	*84.8*	*15.2*
**Path 4**	-445.5	90.9	-470.0	-66.4	*87.6*	*12.4*
**Path 5**	-445.5	89.7	-471.0	-64.2	*88.0*	*12.0*
**Path 6**	-445.5	89.9	-479.1	-56.2	*89.5*	*10.5*
**Path 7**	-445.5	85.8	-487.6	-43.7	*91.8*	*8.2*
**Path 8**	-445.5	80.5	-451.7	-74.2	*85.9*	*14.1*
**Path 9**	-445.5	71.0	-435.4	-81.1	*84.3*	*15.7*
	0.0	*7.1*	*16.6*	*13.4*	*2.8*	*2.8*

Li_2I_2

paths	ΔE_{int}	ΔE_{Pauli}	ΔE_{elstat}	ΔE_{oi}	*%elstat*	*%oi*
**Path 1**	-319.5	58.9	-303.7	-74.7	*80.3*	*19.7*
**Path 2**	-319.5	63.2	-301.7	-81.1	*78.8*	*21.2*
**Path 3**	-319.5	44.3	-255.5	-108.3	*70.2*	*29.8*
**Path 4**	-319.5	63.4	-293.5	-89.4	*76.6*	*23.4*
**Path 5**	-319.5	63.8	-281.6	-101.7	*73.5*	*26.5*
**Path 6**	-319.5	61.3	-304.0	-76.8	*79.8*	*20.2*
**Path 7**	-319.5	58.9	-303.5	-75.0	*80.2*	*19.8*
**Path 8**	-319.5	52.4	-286.7	-85.2	*77.1*	*22.9*
**Path 9**	-319.5	43.2	-274.1	-88.6	*75.6*	*24.4*
	0.0	*6.9*	*18.0*	*13.6*	*3.4*	*3.4*

Cs_2I_2

paths	ΔE_{int}	ΔE_{Pauli}	ΔE_{elstat}	ΔE_{oi}	*%elstat*	*%oi*
**Path 1**	-224.5	71.4	-267.1	-28.9	*90.2*	*9.8*
**Path 2**	-224.5	68.6	-246.6	-46.5	*84.1*	*15.9*
**Path 3**	-224.5	66.5	-245.3	-45.8	*84.3*	*15.7*
**Path 4**	-224.5	70.2	-249.8	-45.0	*84.7*	*15.3*
**Path 5**	-224.5	72.4	-252.6	-44.3	*85.1*	*14.9*
**Path 6**	-224.5	69.9	-255.7	-38.8	*86.8*	*13.2*
**Path 7**	-224.5	71.3	-261.9	-34.0	*88.5*	*11.5*
**Path 8**	-224.5	65.3	-243.7	-46.2	*84.1*	*15.9*
**Path 9**	-224.5	62.4	-241.0	-45.9	*84.0*	*16.0*
	0.0	*2.0*	*8.0*	*6.8*	*2.3*	*2.3*

Be_2O_2

paths	**ΔE_{int}**	**ΔE_{Pauli}**	**ΔE_{elstat}**	**ΔE_{oi}**	***%elstat***	***%oi***
**Path 1**	-2033.5	402.4	-2067.0	-368.9	*84.9*	*15.1*
**Path 2**	-2033.5	487.2	-1934.4	-586.4	*76.7*	*23.3*
**Path 3**	-2033.5	279.7	-1773.9	-539.4	*76.7*	*23.3*
**Path 4**	-2033.5	467.9	-1913.1	-588.3	*76.5*	*23.5*
**Path 5**	-2033.5	480.5	-1914.4	-599.6	*76.1*	*23.9*
**Path 6**	-2033.5	428.0	-1978.7	-482.8	*80.4*	*19.6*
**Path 7**	-2033.5	403.2	-2065.2	-371.5	*84.8*	*15.2*
**Path 8**	-2033.5	341.6	-1698.4	-676.8	*71.5*	*28.5*
**Path 9**	-2033.5	244.9	-1638.5	-640.0	*71.9*	*28.1*
	0.0	*71.6*	*101.4*	*100.4*	*4.8*	*4.8*

Be_2S_2

paths	**ΔE_{int}**	**ΔE_{Pauli}**	**ΔE_{elstat}**	**ΔE_{oi}**	***%elstat***	***%oi***
**Path 1**	-1693.9	312.9	-1405.5	-601.3	*70.0*	*30.0*
**Path 2**	-1693.9	396.6	-1417.0	-673.5	*67.8*	*32.2*
**Path 3**	-1693.9	173.9	-1052.4	-815.4	*56.3*	*43.7*
**Path 4**	-1693.9	360.4	-1347.1	-707.3	*65.6*	*34.4*
**Path 5**	-1693.9	368.4	-1240.8	-821.5	*60.2*	*39.8*
**Path 6**	-1693.9	341.1	-1415.2	-619.8	*69.5*	*30.5*
**Path 7**	-1693.9	313.4	-1405.0	-602.2	*70.0*	*30.0*
**Path 8**	-1693.9	263.0	-1292.3	-664.6	*66.0*	*34.0*
**Path 9**	-1693.9	164.4	-1212.2	-646.2	*65.2*	*34.8*
	0.0	*72.6*	*136.2*	*94.9*	*4.7*	*4.7*

Be_2Se_2

paths	**ΔE_{int}**	**ΔE_{Pauli}**	**ΔE_{elstat}**	**ΔE_{oi}**	***%elstat***	***%oi***
**Path 1**	-1640.9	282.6	-1283.0	-640.5	*66.7*	*33.3*
**Path 2**	-1640.9	379.9	-1324.7	-696.0	*65.6*	*34.4*
**Path 3**	-1640.9	141.5	-907.9	-874.5	*50.9*	*49.1*
**Path 4**	-1640.8	332.2	-1240.9	-732.2	*62.9*	*37.1*
**Path 5**	-1640.9	337.4	-1114.9	-863.4	*56.4*	*43.6*
**Path 6**	-1640.9	318.4	-1312.5	-646.8	*67.0*	*33.0*
**Path 7**	-1640.9	282.9	-1282.7	-641.1	*66.7*	*33.3*
**Path 8**	-1640.9	241.8	-1203.1	-679.6	*63.9*	*36.1*
**Path 9**	-1640.9	139.4	-1112.0	-668.2	*62.5*	*37.5*
	0.0	*76.1*	*150.3*	*102.2*	*5.5*	*5.5*

Be_2Te_2

paths	**ΔE_{int}**	**ΔE_{Pauli}**	**ΔE_{elstat}**	**ΔE_{oi}**	***%elstat***	***%oi***
**Path 1**	-1561.3	247.0	-1099.1	-709.1	*60.8*	*39.2*
**Path 2**	-1561.3	352.7	-1180.4	-733.6	*61.7*	*38.3*
**Path 3**	-1561.3	112.9	-726.5	-947.7	*43.4*	*56.6*
**Path 4**	-1561.2	295.6	-1082.1	-774.7	*58.3*	*41.7*
**Path 5**	-1561.2	298.1	-927.5	-931.8	*49.9*	*50.1*
**Path 6**	-1561.2	288.7	-1157.5	-692.4	*62.6*	*37.4*
**Path 7**	-1561.3	247.2	-1098.9	-709.5	*60.8*	*39.2*
**Path 8**	-1561.3	214.5	-1070.3	-705.4	*60.3*	*39.7*
**Path 9**	-1561.3	114.7	-980.2	-695.8	*58.5*	*41.5*
	0.0	*75.3*	*159.8*	*108.6*	6.4	6.4

Mg_2O_2

paths	**ΔE_{int}**	**ΔE_{Pauli}**	**ΔE_{elstat}**	**ΔE_{oi}**	***%elstat***	***%oi***
**Path 1**	-1691.0	305.7	-1831.0	-165.7	*91.7*	*8.3*
**Path 2**	-1691.0	346.8	-1619.4	-418.4	*79.5*	*20.5*
**Path 3**	-1691.0	255.0	-1694.6	-251.4	*87.1*	*12.9*
**Path 4**	-1691.0	336.1	-1635.6	-391.4	*80.7*	*19.3*
**Path 5**	-1691.0	351.8	-1704.9	-337.9	*83.5*	*16.5*
**Path 6**	-1691.0	311.4	-1684.6	-317.8	*84.1*	*15.9*
**Path 7**	-1691.0	305.7	-1823.9	-172.8	*91.3*	*8.7*
**Path 8**	-1691.0	253.6	-1475.5	-469.1	*75.9*	*24.1*
**Path 9**	-1691.0	207.2	-1483.8	-414.5	*78.2*	*21.8*
	0.0	*33.1*	*83.8*	*100.3*	5.6	5.6

Mg_2S_2

paths	**ΔE_{int}**	**ΔE_{Pauli}**	**ΔE_{elstat}**	**ΔE_{oi}**	***%elstat***	***%oi***
**Path 1**	-1399.5	267.2	-1396.7	-269.9	*83.8*	*16.2*
**Path 2**	-1399.5	307.7	-1277.2	-430.0	*74.8*	*25.2*
**Path 3**	-1399.5	183.0	-1190.1	-392.4	*75.2*	*24.8*
**Path 4**	-1399.5	294.7	-1265.1	-429.1	*74.7*	*25.3*
**Path 5**	-1399.5	313.1	-1266.5	-446.0	*74.0*	*26.0*
**Path 6**	-1399.5	275.2	-1313.8	-360.8	*78.5*	*21.5*
**Path 7**	-1399.5	267.2	-1394.1	-272.6	*83.6*	*16.4*
**Path 8**	-1399.5	230.4	-1217.1	-412.7	*74.7*	*25.3*
**Path 9**	-1399.5	163.1	-1194.9	-367.7	*76.5*	*23.5*
	0.0	*43.7*	*74.5*	*74.1*	3.9	3.9

Mg_2Se_2

paths	ΔE_{int}	ΔE_{Pauli}	ΔE_{elstat}	ΔE_{oi}	*%elstat*	*%oi*
**Path 1**	-1359.5	249.3	-1303.6	-305.2	*81.0*	*19.0*
**Path 2**	-1359.5	298.9	-1208.1	-450.3	*72.8*	*27.2*
**Path 3**	-1359.5	156.4	-1056.9	-459.0	*69.7*	*30.3*
**Path 4**	-1359.5	280.8	-1184.7	-455.6	*72.2*	*27.8*
**Path 5**	-1359.5	297.4	-1171.0	-485.8	*70.7*	*29.3*
**Path 6**	-1359.5	263.4	-1235.8	-387.1	*76.1*	*23.9*
**Path 7**	-1359.5	249.4	-1301.5	-307.4	*80.9*	*19.1*
**Path 8**	-1359.5	218.5	-1147.5	-430.5	*72.7*	*27.3*
**Path 9**	-1359.5	144.0	-1105.1	-398.3	*73.5*	*26.5*
	0.0	*48.7*	*85.1*	*75.1*	*4.1*	*4.1*

Ba_2O_2

paths	ΔE_{int}	ΔE_{Pauli}	ΔE_{elstat}	ΔE_{oi}	*%elstat*	*%oi*
**Path 1**	-1429.3	601.1	-1741.9	-288.6	*85.8*	*14.2*
**Path 2**	-1429.3	560.0	-1504.8	-484.5	*75.6*	*24.4*
**Path 3**	-1429.3	575.0	-1595.6	-408.8	*79.6*	*20.4*
**Path 4**	-1429.3	590.1	-1554.7	-464.8	*77.0*	*23.0*
**Path 5**	-1429.3	625.4	-1646.3	-408.4	*80.1*	*19.9*
**Path 6**	-1429.3	582.8	-1595.9	-416.3	*79.3*	*20.7*
**Path 7**	-1429.3	607.3	-1673.4	-363.2	*82.2*	*17.8*
**Path 8**	-1429.3	488.9	-1236.3	-681.9	*64.5*	*35.5*
**Path 9**	-1429.3	462.6	-1213.9	-678.1	*64.2*	*35.8*
	0.0	*21.7*	*78.5*	*65.0*	*7.5*	*7.5*

Ba_2Se_2

paths	ΔE_{int}	ΔE_{Pauli}	ΔE_{elstat}	ΔE_{oi}	*%elstat*	*%oi*
**Path 1**	-1080.2	478.4	-1351.3	-207.2	*86.7*	*13.3*
**Path 2**	-1080.2	395.9	-1132.1	-344.0	*76.7*	*23.3*
**Path 3**	-1080.2	389.1	-1153.5	-315.8	*78.5*	*21.5*
**Path 4**	-1080.2	434.7	-1183.6	-331.3	*78.1*	*21.9*
**Path 5**	-1080.2	481.6	-1255.0	-306.8	*80.4*	*19.6*
**Path 6**	-1080.2	434.1	-1214.7	-299.6	*80.2*	*19.8*
**Path 7**	-1080.2	477.5	-1326.0	-231.7	*85.1*	*14.9*
**Path 8**	-1080.2	374.4	-1092.7	-361.9	*75.1*	*24.9*
**Path 9**	-1080.2	329.8	-1055.3	-354.7	*74.8*	*25.2*
	0.0	*39.2*	*84.1*	*51.5*	*4.1*	*4.1*

Ba_2Te_2

paths	**ΔE$_{int}$**	**ΔE$_{Pauli}$**	**ΔE$_{elstat}$**	**ΔE$_{oi}$**	***%elstat***	***%oi***
**Path 1**	-1011.3	428.0	-1232.1	-207.3	*85.6*	*14.4*
**Path 2**	-1011.3	349.8	-1033.4	-327.8	*75.9*	*24.1*
**Path 3**	-1011.3	334.8	-1026.4	-319.7	*76.3*	*23.7*
**Path 4**	-1011.3	385.1	-1076.2	-320.2	*77.1*	*22.9*
**Path 5**	-1011.3	429.3	-1134.6	-306.0	*78.8*	*21.2*
**Path 6**	-1011.3	383.7	-1106.9	-288.1	*79.3*	*20.7*
**Path 7**	-1011.3	427.1	-1213.4	-225.0	*84.4*	*15.6*
**Path 8**	-1011.3	331.8	-1009.3	-333.9	*75.1*	*24.9*
**Path 9**	-1011.3	286.4	-970.0	-327.8	*74.7*	*25.3*
	0.0	*38.9*	*81.4*	*48.9*	*3.9*	*3.9*

B_2N_2

paths	**ΔE$_{int}$**	**ΔE$_{Pauli}$**	**ΔE$_{elstat}$**	**ΔE$_{oi}$**	***%elstat***	***%oi***
**Path 1**	-4846.9	1907.6	-4867.2	-1887.3	*72.1*	*27.9*
**Path 2**	-4846.9	1346.3	-3670.7	-2522.5	*59.3*	*40.7*
**Path 3**	-4846.9	1392.6	-3770.7	-2468.8	*60.4*	*39.6*
**Path 4**	-4846.9	1733.8	-3850.4	-2730.3	*58.5*	*41.5*
**Path 5**	-4846.9	1789.0	-4528.1	-2107.8	*68.2*	*31.8*
**Path 6**	-4846.9	1616.7	-4027.3	-2436.3	*62.3*	*37.7*
**Path 7**	-4846.9	1907.6	-4866.4	-1888.1	*72.0*	*28.0*
**Path 8**	-4846.9	1548.3	-3071.4	-3323.9	*48.0*	*52.0*
**Path 9**	-4846.9	1364.5	-2685.2	-3526.2	*43.2*	*56.8*
	0.0	*229.4*	*517.8*	*331.2*	*9.9*	*9.9*

B_2P_2

paths	**ΔE$_{int}$**	**ΔE$_{Pauli}$**	**ΔE$_{elstat}$**	**ΔE$_{oi}$**	***%elstat***	***%oi***
**Path 1**	-4237.0	613.1	-2266.3	-2583.7	*46.7*	*53.3*
**Path 2**	-4237.0	899.3	-2733.4	-2402.8	*53.2*	*46.8*
**Path 3**	-4237.0	200.9	-1205.3	-3232.6	*27.2*	*72.8*
**Path 4**	-4237.0	681.1	-2465.5	-2452.6	*50.1*	*49.9*
**Path 5**	-4237.0	674.0	-1798.1	-3112.9	*36.6*	*63.4*
**Path 6**	-4237.0	690.1	-2634.7	-2292.3	*53.5*	*46.5*
**Path 7**	-4237.0	613.4	-2266.2	-2584.2	*46.7*	*53.3*
**Path 8**	-4237.0	478.1	-2424.1	-2291.0	*51.4*	*48.6*
**Path 9**	-4237.0	213.4	-2192.3	-2258.0	*49.3*	*50.7*
	0.0	*210.2*	*532.9*	*362.5*	*8.7*	*8.7*

B_2As_2

paths	**ΔE$_{int}$**	**ΔE$_{Pauli}$**	**ΔE$_{elstat}$**	**ΔE$_{oi}$**	***%elstat***	***%oi***
**Path 1**	-4194.1	633.6	-2587.2	-2240.5	*53.6*	*46.4*
**Path 2**	-4194.1	777.6	-2653.1	-2318.6	*53.4*	*46.6*
**Path 3**	-4194.1	192.5	-1555.4	-2831.2	*35.5*	*64.5*
**Path 4**	-4194.1	668.7	-2332.6	-2530.2	*48.0*	*52.0*
**Path 5**	-4194.1	703.3	-2133.2	-2764.3	*43.6*	*56.4*
**Path 6**	-4194.1	746.8	-2516.7	-2424.3	*50.9*	*49.1*
**Path 7**	-4194.1	633.7	-2587.1	-2240.7	*53.6*	*46.4*
**Path 8**	-4194.1	578.7	-2396.8	-2376.0	*50.2*	*49.8*
**Path 9**	-4194.1	181.2	-2150.8	-2224.5	*49.2*	*50.8*
	0.0	*197.2*	*389.4*	*241.7*	*5.9*	*5.9*

B_2Sb_2

paths	**ΔE$_{int}$**	**ΔE$_{Pauli}$**	**ΔE$_{elstat}$**	**ΔE$_{oi}$**	***%elstat***	***%oi***
**Path 1**	-4029.3	534.5	-1816.2	-2747.6	*39.8*	*60.2*
**Path 2**	-4029.3	739.5	-2307.8	-2460.9	*48.4*	*51.6*
**Path 3**	-4029.3	151.0	-838.8	-3341.5	*20.1*	*79.9*
**Path 4**	-4029.3	723.2	-2165.0	-2587.5	*45.6*	*54.4*
**Path 5**	-4029.3	716.2	-1474.6	-3270.9	*31.1*	*68.9*
**Path 6**	-4029.3	578.7	-2229.4	-2378.7	*48.4*	*51.6*
**Path 7**	-4029.3	534.7	-1816.2	-2747.8	*39.8*	*60.2*
**Path 8**	-4031.5	390.3	-2053.1	-2368.7	*46.4*	*53.6*
**Path 9**	-4031.5	163.6	-1841.5	-2353.6	*43.9*	*56.1*
	1.0	*204.8*	*517.3*	*378.0*	*9.4*	*9.4*

Ga_2N_2

paths	**ΔE$_{int}$**	**ΔE$_{Pauli}$**	**ΔE$_{elstat}$**	**ΔE$_{oi}$**	***%elstat***	***%oi***
**Path 1**	-4137.2	920.3	-4291.5	-766.0	*84.9*	*15.1*
**Path 2**	-4137.2	818.7	-3451.2	-1504.7	*69.6*	*30.4*
**Path 3**	-4137.2	606.9	-3654.0	-1090.0	*77.0*	*23.0*
**Path 4**	-4137.2	906.6	-3557.6	-1486.2	*70.5*	*29.5*
**Path 5**	-4137.2	1020.7	-4022.8	-1135.1	*78.0*	*22.0*
**Path 6**	-4137.2	837.3	-3671.5	-1303.1	*73.8*	*26.2*
**Path 7**	-4137.2	919.2	-4263.5	-792.9	*84.3*	*15.7*
**Path 8**	-4137.2	751.5	-3110.7	-1777.9	*63.6*	*36.4*
**Path 9**	-4137.2	499.2	-2942.2	-1694.2	*63.5*	*36.5*
	0.0	*130.0*	*344.1*	*300.3*	*7.9*	*7.9*

Ga_2P_2

paths	**ΔE_{int}**	**ΔE_{Pauli}**	**ΔE_{elstat}**	**ΔE_{oi}**	***%elstat***	***%oi***
**Path 1**	-3511.7	686.8	-3051.6	-1147.0	*72.7*	*27.3*
**Path 2**	-3511.7	620.1	-2579.2	-1552.6	*62.4*	*37.6*
**Path 3**	-3511.7	310.3	-2250.3	-1571.7	*58.9*	*41.1*
**Path 4**	-3511.7	716.3	-2583.2	-1644.7	*61.1*	*38.9*
**Path 5**	-3511.7	792.4	-2681.6	-1622.6	*62.3*	*37.7*
**Path 6**	-3511.7	640.9	-2706.8	-1445.7	*65.2*	*34.8*
**Path 7**	-3511.7	687.4	-3043.4	-1155.7	*72.5*	*27.5*
**Path 8**	-3511.7	496.8	-2347.9	-1660.5	*58.6*	*41.4*
**Path 9**	-3511.7	235.4	-2190.6	-1556.5	*58.5*	*41.5*
	0.0	*154.1*	*280.6*	*212.7*	*5.6*	*5.6*

Ga_2As_2

paths	**ΔE_{int}**	**ΔE_{Pauli}**	**ΔE_{elstat}**	**ΔE_{oi}**	***%elstat***	***%oi***
**Path 1**	-3497.9	638.4	-2975.1	-1161.1	*71.9*	*28.1*
**Path 2**	-3497.9	516.1	-2430.4	-1583.6	*60.5*	*39.5*
**Path 3**	-3497.9	277.2	-2202.9	-1572.2	*58.4*	*41.6*
**Path 4**	-3497.9	660.8	-2391.1	-1767.5	*57.5*	*42.5*
**Path 5**	-3497.9	742.5	-2584.1	-1656.3	*60.9*	*39.1*
**Path 6**	-3497.9	583.2	-2521.1	-1560.0	*61.8*	*38.2*
**Path 7**	-3497.9	638.3	-2967.1	-1169.1	*71.7*	*28.3*
**Path 8**	-3497.9	492.4	-2319.1	-1671.1	*58.1*	*41.9*
**Path 9**	-3497.9	235.8	-2191.1	-1542.6	*58.7*	*41.3*
	0.0	*150.3*	*291.5*	*236.7*	*5.7*	*5.7*

In_2N_2

paths	**ΔE_{int}**	**ΔE_{Pauli}**	**ΔE_{elstat}**	**ΔE_{oi}**	***%elstat***	***%oi***
**Path 1**	-3959.5	1941.7	-3532.5	-2368.7	*59.9*	*40.1*
**Path 2**	-3959.5	1414.4	-2936.1	-2437.9	*54.6*	*45.4*
**Path 3**	-3959.5	1647.1	-2983.6	-2623.0	*53.2*	*46.8*
**Path 4**	-3969.0	1793.3	-3191.8	-2570.5	*55.4*	*44.6*
**Path 5**	-3959.5	1848.3	-3728.4	-2079.4	*64.2*	*35.8*
**Path 6**	-3959.5	1631.8	-3169.6	-2421.7	*56.7*	*43.3*
**Path 7**	-3959.5	1935.9	-3495.2	-2400.2	*59.3*	*40.7*
**Path 8**	-3959.5	1543.6	-2491.8	-3011.3	*45.3*	*54.7*
**Path 9**	-3959.5	1606.8	-2039.1	-3527.2	*36.6*	*63.4*
	3.2	*191.4*	*299.0*	*174.6*	*8.3*	*8.3*

In_2P_2

paths	ΔE_{int}	ΔE_{Pauli}	ΔE_{elstat}	ΔE_{oi}	*%elstat*	*%oi*
**Path 1**	-3242.3	749.2	-3066.5	-925.0	*76.8*	*23.2*
**Path 2**	-3242.3	566.1	-2427.5	-1380.9	*63.7*	*36.3*
**Path 3**	-3242.3	396.6	-2358.3	-1280.6	*64.8*	*35.2*
**Path 4**	-3242.3	731.2	-2460.3	-1513.2	*61.9*	*38.1*
**Path 5**	-3242.3	847.9	-2708.2	-1382.0	*66.2*	*33.8*
**Path 6**	-3242.3	662.9	-2572.2	-1333.0	*65.9*	*34.1*
**Path 7**	-3242.3	746.8	-3042.9	-946.2	*76.3*	*23.7*
**Path 8**	-3242.3	560.9	-2337.7	-1465.5	*61.5*	*38.5*
**Path 9**	-3242.3	316.6	-2223.9	-1335.0	*62.5*	*37.5*
	0.0	*149.0*	*290.6*	*227.1*	5.9	5.9

In_2As_2

paths	ΔE_{int}	ΔE_{Pauli}	ΔE_{elstat}	ΔE_{oi}	*%elstat*	*%oi*
**Path 1**	-3232.3	695.4	-2980.5	-947.2	*75.9*	*24.1*
**Path 2**	-3232.3	533.8	-2366.4	-1399.7	*62.8*	*37.2*
**Path 3**	-3232.3	365.3	-2299.8	-1297.9	*63.9*	*36.1*
**Path 4**	-3232.3	680.9	-2373.0	-1540.2	*60.6*	*39.4*
**Path 5**	-3232.3	788.1	-2609.1	-1411.3	*64.9*	*35.1*
**Path 6**	-3232.3	619.2	-2494.3	-1357.2	*64.8*	*35.2*
**Path 7**	-3232.3	692.6	-2957.9	-967.0	*75.4*	*24.6*
**Path 8**	-3232.3	494.7	-2216.0	-1510.9	*59.5*	*40.5*
**Path 9**	-3232.3	265.9	-2119.7	-1378.5	*60.6*	*39.4*
	0.0	*138.5*	*282.4*	*228.8*	6.1	6.1

Tl_2N_2

paths	ΔE_{int}	ΔE_{Pauli}	ΔE_{elstat}	ΔE_{oi}	*%elstat*	*%oi*
**Path 1**	-4093.7	1968.9	-3289.6	-2773.0	*54.3*	*45.7*
**Path 2**	-4093.7	1448.4	-2805.2	-2736.9	*50.6*	*49.4*
**Path 3**	-4093.7	1719.1	-2809.6	-3003.2	*48.3*	*51.7*
**Path 4**	-4093.7	1802.8	-3072.5	-2824.0	*52.1*	*47.9*
**Path 5**	-4093.7	1845.8	-3573.7	-2365.8	*60.2*	*39.8*
**Path 6**	-4093.7	1644.4	-3013.6	-2724.5	*52.5*	*47.5*
**Path 7**	-4093.7	1962.3	-3243.4	-2812.6	*53.6*	*46.4*
**Path 8**	-4093.7	1568.4	-2365.7	-3296.4	*41.8*	*58.2*
**Path 9**	-4093.7	1686.2	-1895.3	-3884.6	*32.8*	*67.2*
	0.0	*184.9*	*276.5*	*192.5*	8.0	8.0

Tl_2As_2

paths	**ΔE$_{int}$**	**ΔE$_{Pauli}$**	**ΔE$_{elstat}$**	**ΔE$_{oi}$**	***%elstat***	***%oi***
**Path 1**	-3338.6	934.7	-3254.8	-1018.5	*76.2*	*23.8*
**Path 2**	-3338.6	600.3	-2433.3	-1505.5	*61.8*	*38.2*
**Path 3**	-3338.6	509.0	-2489.6	-1357.9	*64.7*	*35.3*
**Path 4**	-3338.6	845.9	-2558.3	-1612.1	*61.3*	*38.7*
**Path 5**	-3338.6	977.9	-2967.5	-1334.9	*69.0*	*31.0*
**Path 6**	-3338.6	755.8	-2652.4	-1442.0	*64.8*	*35.2*
**Path 7**	-3338.6	929.7	-3227.7	-1040.5	*75.6*	*24.4*
**Path 8**	-3338.6	660.1	-2378.2	-1620.4	*59.5*	*40.5*
**Path 9**	-3338.6	412.2	-2181.7	-1569.1	*58.2*	*41.8*
	0.0	*180.2*	*348.5*	*225.3*	*6.6*	*6.6*

Ag_2Cl_2

paths	**ΔE$_{int}$**	**ΔE$_{Pauli}$**	**ΔE$_{elstat}$**	**ΔE$_{oi}$**	***%elstat***	***%oi***
**Path 1**	-381.5	182.2	-456.4	-107.4	*80.9*	*19.1*
**Path 2**	-381.5	205.9	-431.2	-156.3	*73.4*	*26.6*
**Path 3**	-381.5	182.0	-420.6	-143.0	*74.6*	*25.4*
**Path 4**	-381.5	204.6	-428.9	-157.3	*73.2*	*26.8*
**Path 5**	-381.6	213.1	-426.5	-168.1	*71.7*	*28.3*
**Path 6**	-381.5	191.2	-438.4	-134.4	*76.5*	*23.5*
**Path 7**	-381.6	192.8	-446.9	-127.4	*77.8*	*22.2*
**Path 8**	-381.5	177.7	-414.5	-144.7	*74.1*	*25.9*
**Path 9**	-381.5	162.4	-416.0	-127.9	*76.5*	*23.5*
	0.0	*12.1*	*12.5*	*20.8*	*2.8*	*2.8*

Ag_2Br_2

paths	**ΔE$_{int}$**	**ΔE$_{Pauli}$**	**ΔE$_{elstat}$**	**ΔE$_{oi}$**	***%elstat***	***%oi***
**Path 1**	-374.9	198.2	-454.3	-118.9	*79.3*	*20.7*
**Path 2**	-374.9	220.6	-433.6	-161.9	*72.8*	*27.2*
**Path 3**	-374.9	189.5	-407.4	-157.0	*72.2*	*27.8*
**Path 4**	-374.9	220.9	-430.9	-164.9	*72.3*	*27.7*
**Path 5**	-374.9	230.9	-426.5	-179.3	*70.4*	*29.6*
**Path 6**	-374.9	206.9	-439.7	-142.1	*75.6*	*24.4*
**Path 7**	-374.9	207.9	-447.5	-135.3	*76.8*	*23.2*
**Path 8**	-374.9	190.0	-414.2	-150.7	*73.3*	*26.7*
**Path 9**	-374.9	169.8	-408.4	-136.3	*75.0*	*25.0*
	0.0	*14.4*	*15.3*	*20.4*	*2.7*	*2.7*

Ag_2I_2

paths	ΔE_{int}	ΔE_{Pauli}	ΔE_{elstat}	ΔE_{oi}	*%elstat*	*%oi*
**Path 1**	-369.5	210.0	-442.8	-136.6	*76.4*	*23.6*
**Path 2**	-369.5	236.8	-431.9	-174.3	*71.2*	*28.8*
**Path 3**	-369.5	190.0	-381.3	-178.1	*68.2*	*31.8*
**Path 4**	-369.5	235.5	-425.8	-179.2	*70.4*	*29.6*
**Path 5**	-369.5	245.0	-416.5	-198.0	*67.8*	*32.2*
**Path 6**	-369.5	220.9	-434.3	-156.1	*73.6*	*26.4*
**Path 7**	-369.5	218.5	-438.5	-149.4	*74.6*	*25.4*
**Path 8**	-369.5	200.7	-407.6	-162.6	*71.5*	*28.5*
**Path 9**	-369.5	173.9	-393.3	-150.1	*72.4*	*27.6*
	0.0	*18.8*	*20.9*	*21.0*	*2.8*	*2.8*

Hg_2O_2

paths	ΔE_{int}	ΔE_{Pauli}	ΔE_{elstat}	ΔE_{oi}	*%elstat*	*%oi*
**Path 1**	-1798.6	387.4	-1786.7	-399.2	*81.7*	*18.3*
**Path 2**	-1798.6	406.4	-1478.2	-726.8	*67.0*	*33.0*
**Path 3**	-1798.6	375.4	-1644.0	-530.0	*75.6*	*24.4*
**Path 4**	-1798.6	403.8	-1501.6	-700.8	*68.2*	*31.8*
**Path 5**	-1798.6	450.7	-1587.3	-662.0	*70.6*	*29.4*
**Path 6**	-1798.6	386.2	-1556.5	-628.3	*71.2*	*28.8*
**Path 7**	-1798.6	403.1	-1717.3	-484.4	*78.0*	*22.0*
**Path 8**	-1798.6	347.7	-1400.8	-745.5	*65.3*	*34.7*
**Path 9**	-1798.6	301.4	-1470.4	-629.5	*70.0*	*30.0*
	0.0	*24.4*	*112.8*	*121.6*	*5.4*	*5.4*

Hg_2S_2

paths	ΔE_{int}	ΔE_{Pauli}	ΔE_{elstat}	ΔE_{oi}	*%elstat*	*%oi*
**Path 1**	-1568.3	495.0	-1614.2	-449.0	*78.2*	*21.8*
**Path 2**	-1568.3	479.3	-1359.7	-687.8	*66.4*	*33.6*
**Path 3**	-1568.3	396.9	-1379.9	-585.2	*70.2*	*29.8*
**Path 4**	-1568.3	505.5	-1381.7	-692.0	*66.6*	*33.4*
**Path 5**	-1568.3	571.5	-1432.3	-707.6	*66.9*	*33.1*
**Path 6**	-1568.3	484.5	-1438.8	-614.0	*70.1*	*29.9*
**Path 7**	-1568.3	508.1	-1581.8	-494.6	*76.2*	*23.8*
**Path 8**	-1568.3	418.8	-1334.8	-652.4	*67.2*	*32.8*
**Path 9**	-1568.3	321.7	-1337.8	-552.2	*70.8*	*29.2*
	0.0	*51.7*	*101.9*	*101.6*	*4.3*	*4.3*
